# Effect of Benzothiadiazole on the Metabolome of Tomato Plants Infected by Citrus Exocortis Viroid

**DOI:** 10.3390/v11050437

**Published:** 2019-05-14

**Authors:** María Pilar López-Gresa, Celia Payá, Ismael Rodrigo, José María Bellés, Susana Barceló, Young Hae Choi, Robert Verpoorte, Purificación Lisón

**Affiliations:** 1 Instituto de Biología Molecular y Celular de Plantas, Universitat Politècnica de València (UPV)-Consejo Superior de Investigaciones Científicas (CSIC), Ciudad Politécnica de la Innovación (CPI), Ingeniero Fausto Elio s/n, 46022 Valencia, Spain; cepamon@etsiamn.upv.es (C.P.); irodrig@ibmcp.upv.es (I.R.); jmbelles@btc.upv.es (J.M.B.); plison@ibmcp.upv.es (P.L.); 2 Department of Applied Statistics and Operational Research, and Quality. Universitat Politècnica de València (UPV), 46022 Valencia, Spain; sbarcelo@eio.upv.es; 3 Natural Products Laboratory, Institute of Biology, Leiden University, 2333 BE Leiden, The Netherlands; y.h.choi@biology.leidenuniv.nl (Y.H.C.); verpoort@chem.leidenuniv.nl (R.V.); 4 College of Pharmacy, Kyung Hee University, Seoul 02447, Korea

**Keywords:** NMR, metabolomics, tomato, viroid, BTH, defence, NahG plants, GABA

## Abstract

Benzothiadiazole (BTH) is a functional analogue of the phytohormone salycilic acid (SA) involved in the plant immune response. NahG tomato plants are unable to accumulate SA, which makes them hypersusceptible to several pathogens. Treatments with BTH increase the resistance to bacterial, fungal, viroid, or viral infections. In this study, metabolic alterations in BTH-treated Money Maker and NahG tomato plants infected by citrus exocortis viroid (CEVd) were investigated by nuclear magnetic resonance spectroscopy. Using multivariate data analysis, we have identified defence metabolites induced after viroid infection and BTH-treatment. Glycosylated phenolic compounds include gentisic and ferulic acid accumulated in CEVd-infected tomato plants, as well as phenylalanine, tyrosine, aspartate, glutamate, and asparagine. Besides, an increase of γ-aminobutyric acid (GABA), glutamine, adenosine, and trigonelline, contributed to a clear discrimination between the metabolome of BTH-treated tomato leaves and their corresponding controls. Among them, GABA was the only metabolite significantly accumulated in both genotypes after the chemical treatment. In view of these results, the addition of GABA was performed on tomato plants infected by CEVd, and a reversion of the NahG hypersusceptibility to CEVd was observed, indicating that GABA could regulate the resistance to CEVd induced by BTH.

## 1. Introduction

Plants respond to biotic stress using both innate and acquired immune systems. When challenged, the resistance proteins (R) of the plants recognize the specific avirulence factors (Avr) derived from the pathogen, triggering a hypersensivity response (HR) in the infected tissues. Following the local pathogen exposure, the distal defensive plant response, known as systemic acquired resistance (SAR), is activated via salicylic acid (SA), inducing the pathogenesis-related (PR) proteins and/or phytoalexins in the uninfected tissues [1]. In contrast, when the acquired immunity is not established, the 5-hydroxylation of SA yields gentisic acid (GA), being the accumulation characteristic of the compatible plant-pathogen interactions [2].

Tomato plants (*Solanum lycopersicum*) inoculated with the citrus exocortis viroid (CEVd) accumulate high levels of both SA and GA, constituting an excellent plant-pathogen system to study the signaling function of these phenolic compounds [3]. In addition, studies carried out with the SA-deficient NahG transgenic tomato plants [4] highlight the possible defensive role of these signaling molecules in the viroid infection [5].

**Figure 1:**
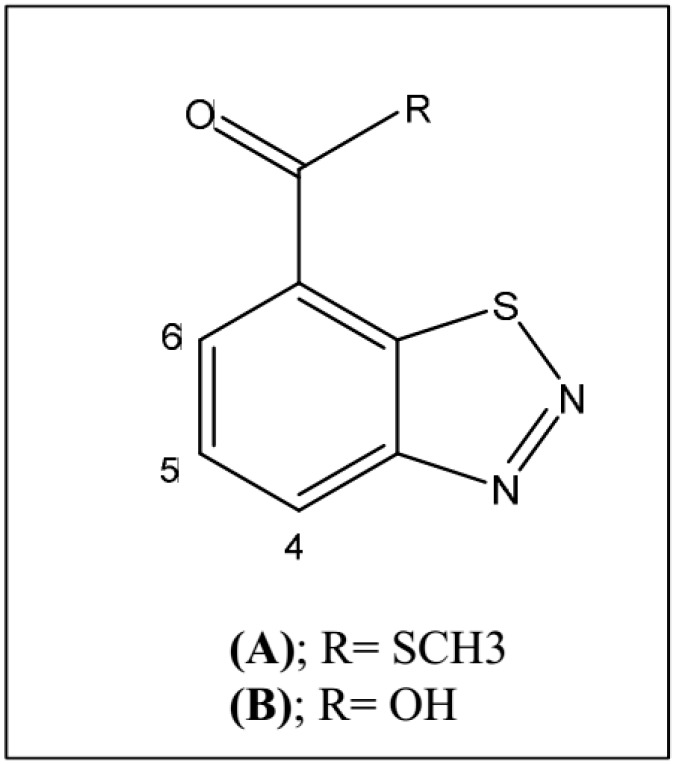
Chemical structures of BTH (**A**) and hydrolyzed BTH (**B**).

Benzo (l,2,3) thiadiazole-7-carbothioic acid-*S*-methyl ester (BTH; Figure 1A), a functional analogue of the plant endogenous hormone SA, is an SAR activator which acts downstream of the SA signaling, providing protection in many crops [6]. Previous work had demonstrated that BTH was a potent inductor of pathogenesis-related genes, thus priming the plants for potential pathogenic attacks [7,8]. Specifically, foliar BTH spray applications of tomato plants conferred resistance against fungal and bacterial infections [9,10]. Moreover, the addition of BTH improves the resistance of the hyper susceptible NahG tomato plants to CEVd and tomato spotted wilt virus (TSWV) infections [5]. BTH applications also produce physiological, biochemical and proteomic changes. In this sense, proteins involved in stress response, energy metabolism, primary and secondary metabolism, signal transduction, and transporters were induced by BTH treatments on muskmelon fruits [11]. However, few reports have been published on how BTH affects the plant metabolome [12]. The activation of key genes involved in secondary metabolic pathways such as phenylalanine ammonia-lyase (PAL), a pivotal gen related to phenylpropanoid metabolism involved in biotic and abiotic stress [13], has been found in cultured parsley cells after the application of both elicitor and BTH [14]. An increase in the levels of stilbenes and anthocyanins in grapevine following BTH treatments has been reported, improving resistance to *Botrytis cinerea* infections [15]. In contrast, the phenolic content was significantly reduced in BTH-treated *Arabidopsis* plants [12].

The employment of the metabolomics platform has provided a new dimension in various study of life sciences, particularly plant-pathogen interactions. This analytical approach enables the monitoring of metabolic alterations produced by different biotic agents. Nuclear magnetic resonance (NMR) is one of the most universally used tools as an analytical platform of metabolomics because it can show the major biomarkers involved in the plant defence response. ^1^H-NMR spectroscopy detects simultaneously the most abundant compounds, offering a macroscopic view of the biochemical situation in the cell at a given point in time [16]. Particularly, studies of *Solanum lycopersicum* infected by different pathogens as viroids, bacteria [17], or viruses [18] showed a large number of primary metabolites differentially accumulated, including amino acids, carbohydrates and organic acids, and some secondary compounds such as phenylpropanoids and flavonoids.

Using these technologies, we study the metabolomic changes in NahG tomato plants, which are not able to accumulate SA, and their corresponding parental Money Maker upon infection with CEVd and BTH treatments. In this work, NMR spectroscopy and partial least square analysis (PLS) were applied to distinguish the induced compounds. The obtained results not only provide information about the metabolic plant adaptation to this chemical, but also about its mechanism of action. Among all the metabolites induced by BTH in tomato plants, we reasoned that GABA, a non-protein, free amino acid involved in multiple signaling processes of abiotic [19] and biotic stresses [20,21,22], could play an important role in the plant defence response of tomato plants to CEVd. To test this hypothesis, the effect of exogenous addition of GABA on tomato defence response and its resistance to CEVd was investigated.

## 2. Materials and Methods

### 2.1. Tomato Plants and Growth Conditions

Tomato plants (*Solanum lycopersicum* cv. Money Maker) and its transformant 35S::NahG expressing the bacterial salicylate hydroxylase [4,23] (kindly provided by Professor J.D.G. Jones, John Innes Centre, Norwich, UK), were used in this study. The tomato seeds were sterilized with a 1:1 mixture of commercial sodium hypochlorite and distilled H_2_O containing a few drops of Tween 20. After the sterilization treatment, they were subsequently sequentially washed with distilled H_2_O during 5, 10, and 15 min. Then, the seeds were transferred to 14 cm diameter Petri dishes with a filter paper soaked with distilled water and were kept in darkness at 24 °C for 48 h, followed by another 48 h in the presence of light. Seeds were placed in 12 cm-diameter pots containing a 1:1 mixture of vermiculite and peat, and were grown under greenhouse conditions with a day/night photoperiod of 16/8 h (30/26 °C), supplemented with artificial light and a relative humidity between 50–70%. The growth conditions were described in detail in a previous work [5].

### 2.2. CEVd Infection Procedure

To study the metabolic changes produced by BTH, a total of twelve Money Maker and twelve NahG plants were used for the experiment. To confirm the protective effect of GABA treatments, eighteen tomato plants of each genotype were used. Half of the plants were infected with CEVd (GenBank accession number S67446) obtained from CEVd-infected Rutgers tomato plants bearing strong symptoms. Inoculation was carried out by rubbing with carborundum, both the first leaf and a cotyledon of 2 week-old plantlets in the presence of 50 ng of the pathogen, as described in López-Gresa et al. [5]. The remaining tomato plants were mock-inoculated by applying 50 μL of sterile demineralized water instead of viroid inoculum. After inoculation, plants were grown under the greenhouse conditions described above. Disease severity was recorded at different time points by measuring all the dwarfing, rugosity, rigidity, and epinasty grade. The tip tissues from mock and CEV-inoculated plants were sampled for the analytical measurements at 2.5- and 3-weeks post infection (wpi), frozen in liquid nitrogen, homogenized in cold conditions, and stored at −80 °C until used.

### 2.3. Treatments with BTH and GABA

Exogenous treatments of GABA and BTH were carried out following the procedure described in López Gresa et al. [5]. A 1 mM solution supplemented with 0.05% (*v*/*v*) Tween 20, was sprayed at 3 and 6 days after viroid inoculation. BTH (Acibenzolar-S-methyl) was supplied as Bion 50WG (50%, *w*/*w*) (Syngenta España, Madrid, Spain), and GABA (γ-aminobutyric acid) was purchased from Sigma-Aldrich (Madrid, Spain).

To perform the NMR metabolomics study after CEVd infection, one half of the Money Maker and NahG plants were sprayed with 1 mM BTH, and the other half was treated with 0.05% (*v*/*v*) Tween 20 in demineralized water at the same time points.

To analyze the primary metabolite content after BTH treatments, two groups of six Money Maker and six NahG 4-week-old tomato plants were used. Three control plants of each group were sprayed with 0.05% (*v*/*v*) Tween 20 in demineralized water and the others were sprayed with 1 mM BTH. Fifth and sixth tomato leaves were harvested at 48 h post-treatment (hpt) and immediately frozen in liquid nitrogen and stored at −80 °C until processed for GC-MS analysis.

To study the protective effect of GABA, two groups of eighteen Money Maker and eighteen NahG plants were prepared. One group of six tomato plants in each variety was treated with 0.05% (*v*/*v*) Tween 20 in demineralized water, and the second and third groups were sprayed with 1 mM BTH or 1 mM GABA solutions, respectively.

### 2.4. Extraction of Metabolites and NMR Spectra Measurements

For the analysis of polar and semi-polar metabolites, 25 mg of freeze-dried plant material (3 wpi) were extracted in 2 mL-Eppendorf tubes with 1 mL of a 1:1 mixture of KH_2_PO_4_ buffer (pH 6) in D_2_O containing 0.05% trimethylsilane propionic acid sodium salt (TMSP) and tetradeuteromethanol (CH_3_OH-d_4_). The mixture was vortexed at room temperature for 1 min, sonicated for 20 min, and centrifuged at 13,000 rpm at room temperature for 10 min. A volume of 700 µL of the supernatant was transferred to a 5 mm-NMR tube for the spectral analysis. ^1^H-NMR, 2D-J resolved, and ^1^H–^1^H correlated spectroscopy (COSY) were recorded at 25 °C on a 600 MHz Bruker AV 600 spectrometer equipped with cryoprobe operating at a proton NMR frequency of 600.13 MHz. As internal lock, methyl signals of CH_3_OH-d_4_ were used. Each ^1^H-NMR spectrum consisted of 128 scans requiring a 10 min acquisition time with the following parameters: 0.25 Hz/point, pulse width of 30° (10.8 μs), and relaxation delay of 1.5 s. To suppress the residual H_2_O signal, a pre-saturation sequence was used at the H_2_O frequency during the recycle delay. Free induction decays were Fourier transformed with line broadening (0.3 Hz) and the spectra were zero-filled to 32 K points. The resulting spectra were manually phased, baseline-corrected, and calibrated to TMSP at 0.0 ppm, using Topspin version 2.1, (Bruker, Billerica, MA, USA).

### 2.5. RNA Extraction and Preparation

Total RNA of tomato leaf tissue of 2.5 wpi plants was isolated using TRIzol reagent (Invitrogen, Carlsbad, CA, USA) according to the manufacturer’s protocol. RNA was further precipitated by adding one volume of LiCl 6 M, and then the pellet was washed with LiCl 3 M and dissolved in RNase-free water. To remove contaminating genomic DNA, TURBO DNase (Ambion, Carlsbad, CA, USA) were added (2 units/10 µg of RNA).

### 2.6. Quantitative Real-Time Polymerase Chain Reaction (PCR) Assay

To obtain the corresponding cDNA target sequences, one µg of total RNA was retrotranscribed using an oligo (dT)_18_ primer and the Prime Script RT reagent kit (Perfect Real Time, Takara, Kusatsu, Shiga, Japan). PCR was performed in the presence of the double-stranded DNA-specific dye Power SYBR Green PCR Master Mix (Applied Biosystems, Carlsbad, CA, USA). Amplification was monitored in real time with the 7500 FAST Real-Time PCR System (Life Technologies, Carlsbad, CA, USA). The PCR primers used for RT-PCRs are listed in Table S1.

### 2.7. GC-MS Analysis for Primary Metabolite

Primary metabolite analysis was performed at the Metabolomics Platform of the Instituto de Biología Molecular y Celular de Plantas (UPV-CSIC, Valencia, Spain) using a method modified from that described by Roessner [24]. One hundred mg of frozen tomato leaves per sample were homogeneized with liquid nitrogen and extracted in 1400 µL 100% methanol containing 60 µL of internal standard (0.2 mg/mL ribitol). The mixture was kept for 15 min at 70 °C; subsequently, the extract was centrifuged for 10 min at 14,000 rpm. Supernatant was transferred to a glass vial where 750 µL of CHCl_3_ and 1500 µL of water were added. The mixture was vortexed for 15 s and centrifuged for 15 min at 14,000 rpm. 150 µL aliquots of the methanol/water supernatant were dried in a vacuum for 6–16 h.

For derivatisation, dry residues were re-dissolved in 40 µL of 20 mg/mL methoxyamine hydrochloride prepared in pyridine and incubated for 90 min at 37 °C, followed by the addition of 70 µL MSTFA (*N*-methyl-*N*-[trimethylsilyl]trifluoroacetamide) and 6 µL of a retention time standard mixture (3.7% [*w*/*v*] mix of fatty acid methyl esters ranging from C8 to C24) and further incubation for 30 min at 37 °C.

Sample volumes of 2 µL were injected in split and spitless mode to increase metabolite detection range in a 6890N gas chromatograph (Agilent Technologies, Santa Clara, CA, USA) coupled to a Pegasus 4D TOF LECO mass spectrometer. Gas chromatography was performed on a BPX35 (30 m × 0.32 mm × 0.25 μm) column with helium as carrier gas at a constant flow of 2 mL/min. The liner was set at 230 °C. Oven program was 85 °C for 2 min, 8 °C/min ramp until 360 °C. Mass spectra were collected at 6.25 spectra·s^−1^ in the m/z range 35–900 and ionization energy of 70 eV. Chromatograms and mass spectra were evaluated using the ChromaTOF software (LECO Corporation, Saint Joseph, MI, USA). The unequivocal compound identification of the 65 primary metabolites was carried out by comparing both mass spectra and retention times with those of pure standards. All the commercial standards were purchased from Sigma–Aldrich (Madrid, Spain).

### 2.8. Statistical Analysis

For the untargeted NMR-metabolomics analysis, the ^1^H-NMR spectra were automatically reduced by AMIX (v. 3.7, Bruker Biospin, Billerica, MA, USA) to ASCII files. Spectral intensities were scaled to total intensity TMSP and reduced to integrated regions of equal width (0.04 ppm) corresponding to the region of δ 0.4–10.00. The regions of δ 4.70–4.90 and δ 3.28–3.34 were excluded from the analysis because of the residual signals of water and methanol, respectively. Partial least square (PLS) and orthogonal projection to latent structures-discriminant analysis (OPLS-DA) were performed with the SIMCA-P software (v. 13.0.3, Umetrics, Umeå, Sweden) using the Pareto scaling method. For the targeted GC-MS-metabolomics analysis, the area of each primary metabolite relative to ribitol area was used as the X variable in the Principal Component Analysis (PCA) using unit variance (UV) scaling method.

To compare the symptoms between non-treated and chemical treated CEVd-infected NahG tomato plants, the symptom severity was monitored and scored at every time point and statistically analyzed by a Kruskal-Wallis test (non-parametric test equivalent to the one-way ANOVA). Different letters indicate significant differences (*p* < 0.05) between non-treated and chemical treated CEVd-infected NahG tomato plants.

For the qRT-PCR analysis, a *t*-test analysis was performed. Comparisons between multiple groups were made by analysis of variance (ANOVA) for each time point. A *p* value < 0.05 was considered significant.

The IBM SPSS v.19 package was used throughout the statistical analysis.

## 3. Results

### 3.1. Partial Least Square of ^1^H-NMR Spectra of Control and BTH-treated Money Maker and NahG Tomato Plants Infected by CEVd and Mock-Inoculated

Based on previous knowledge about tomato leaf tissue, a new ^1^H-NMR study was performed to identify the metabolic changes after BTH treatments on NahG and Money Maker plants after CEVd inoculation. The analysis of the one- and two-dimensional spectra of the plant extracts, along with our in-house database of standards and published data in our previous studies, allowed for the identification of several compounds described in tomato leaves.

**Figure 2:**
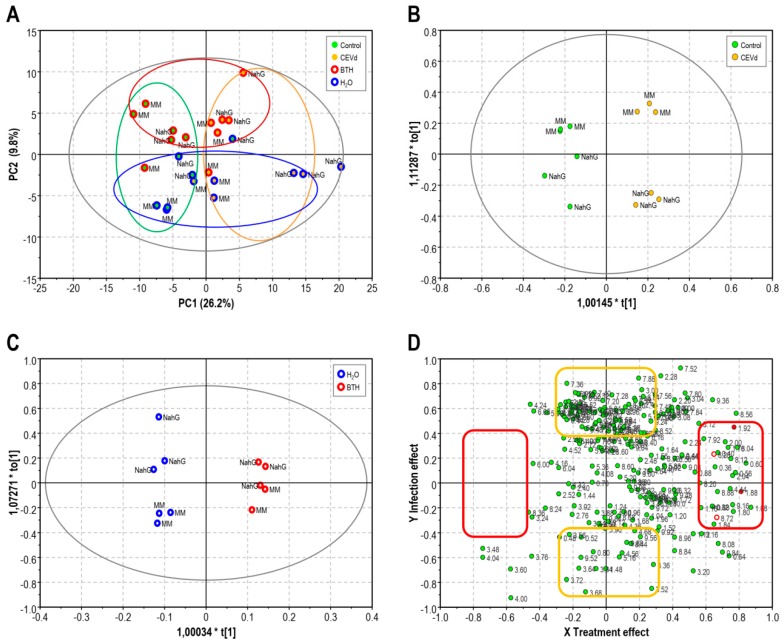
Multivariable data analysis based on the ^1^H-NMR signals, in the range of 0.3–10.0 ppm, of the Money Maker (MM) and NahG tomato plants after viroid inoculation and BTH treatments at 3 weeks post infection (green; mock tomato leaves, yellow; tomato leaves infected by CEVd, red; leaves after BTH treatments blue; leaves after H_2_O treatments). (**A**) Score plot of partial least square (PLS) (**B**) Score plot of orthogonal projection to latent structures-discriminant analysis (OPLS-DA) of the infection (*R*^2^ = 0.95, *Q*^2^ = 0.82). (**C**) Score plot of OPLS-DA of chemical treatment (*R*^2^ = 0.99, *Q*^2^ = 0.93). (**D**) Shared-and-unique-structures (SUS) plot correlating the two OPLS-DA models with the Y axis as CEVd infection and X axis as BTH treatment (^1^H-NMR signals of GABA in red full circles and ^1^H-NMR signals of hydrolyzed BTH in red open circles).

After the chemical analysis of the spectra, a comparison of all the samples involved in the metabolomic study was performed through multivariate data analysis (MVDA). Specifically, a PLS analysis was applied, defining as the X variable the area of binned ^1^H-NMR signals of plant extracts at 3 wpi, and as the step-wise Y variables the genotype (Money Maker and NahG), the treatment (BTH and H_2_O), and the infection (CEVd and mock inoculation). A clear separation was observed in the score plot of PLS (Figure 2A) between the healthy control and CEVd infected plants by component 1, and BTH or H_2_O treatments by component 2. However, no separation was observed in the PLS score plot among Money Maker and NahG plants, indicating that the metabolic content of both cultivars did not differ as much as after the infection and treatments. In fact, both CEVd-inoculated genotypes were in the positive part of PLS-component 1, and both BTH-treated genotypes were in the positive part of PLS-component 2 independently of the chemical treatment or the infection, respectively.

Due to the large number of variables, the PLS could not clearly reveal the metabolites accumulated after the BTH treatment. Therefore, two orthogonal projections to latent structures-discriminant analyses (OPLS-DA) were applied separately to specifically examine the metabolic changes produced by the infection (healthy-CEVd infected; Figure 2B) or the chemical action (H_2_O-BTH; Figure 2C). In the score plot of both OPLS-DA models, the samples inoculated by CEVd or sprayed with BTH were clearly separated from their respective controls. These two OPLS-DA models (infection and chemical treatment) were integrated by a shared-and-unique-structures (SUS)-plot (Figure 2D) with the X-axis corresponding to BTH-treatment and the Y-axis to CEVd infection. In this analysis, the diagonally-aligned metabolites were of equal importance and shared by the two models, and the metabolites located along the axis were specifically altered in CEVd-infected (yellow boxes) or BTH-treated tomato plants (red boxes), respectively.

When comparing the metabolome of the control and infected leaves (positive side of *y*-axis SUS-plot yellow boxed, Figure 2D), it was observed that glycosylated phenolic compounds such as gentisic and ferulic acids, as well as the amino acids phenylalanine, tyrosine, aspartate, glutamate, and asparagine were induced in the viroid infection.

The inducing effect of BTH was characterized by the positive side of the x-axis (right side of x-axis SUS-plot red boxed, Figure 2D) in which GABA, glutamic acid, adenosine, trigonelline, in addition to BTH and its catabolized forms, were found to be the major contributing metabolites induced by the chemical treatment. In fact, the signals observed in the ^1^H-NMR spectra at δ 8.72 (H-6, dd, 7.5, 2.3 Hz), δ 8.28 (H-4, d, 7.5 Hz), and δ 7.84 (H-5, t, 7.5 Hz) were discriminant in the SUS-plot model, and were assigned to the residual hydrolyzed BTH (Figure 1B) in accordance with previous results in BTH-treated Arabidopsis plants [12].

To better understand the differences between Money Maker and NahG plants, a *t*-test analysis comparing a single variable (BTH) was performed using the ^1^H-NMR binned data of each genotype, in both mock-inoculated and CEVd inoculated plants. By doing so, hydrolyzed-BTH and GABA were the compounds that significantly accumulated in mock BTH-treated Money Maker plants, with respect to the corresponding mock non-treated Money Maker plants, whereas GABA and trigonelline displayed higher levels in BTH-treated NahG plants (Table 1).

Moreover, when comparing Money Maker and NahG plants after chemical treatments, the levels of GABA were higher in the transgenic plants, compared to the corresponding non-treated tomato plants. GABA was the most prominent metabolite differentially accumulated in all the comparisons, thus indicating that this molecule could play an important role in the BTH-mediated response. Interestingly, GABA was also significatively accumulated in CEVd-infected and non- treated NahG plants, with respect to the corresponding non infected plants, no detecting differences caused by the viroid infection in the Money Maker parental plants.

On the other hand, trigonelline, malic, and aspartic acids were induced by BTH in Money Maker infected plants. The same compounds together with α-glucose, citric acid, the amino acids alanine and threonine, and the phenylpropanoids caffeic and ferulic acids were found to accumulate in the infected NahG plants after BTH treatment, with respect to the non-treated transgenic infected plants. Besides, both phenolics together with citric and malic acids also accumulated differentially in BTH treated and infected NahG plants, with respect to the corresponding parental plants. The defensive role of these phenolics could explain their accumulation in the transgenic tomato plants, which recovered from their hyper susceptibility phenotype to viroid infection when treated with BTH [25].

### 3.2. Study of the Relative Expression Levels of Tomato Genes Involved in GABA Synthesis in Control and BTH-Treated Money Maker and NahG Tomato Plants

**Figure 3:**
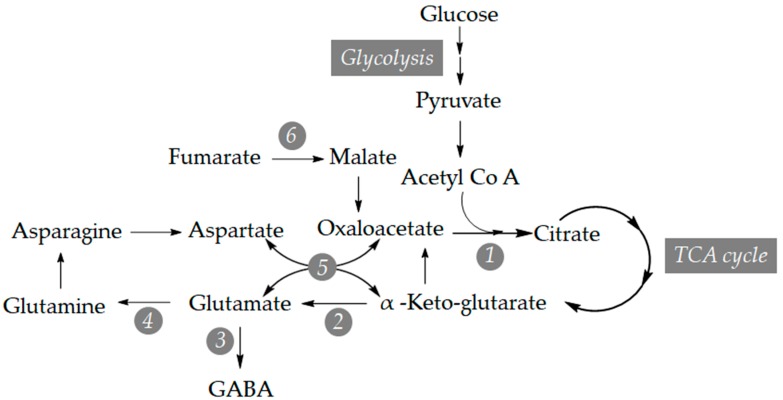
The metabolic inter-relationship between plant metabolism of malate, aspartate, GABA and glucose. **1**: Citrate synthase (mCS), **2**: Glutamate dehydrogenase (GDH), **3**: Glutamate decarboxylase (GAD), **4**: Glutamine synthetase (GS), **5**: Aspartate aminotransferase (AST), **6**: Fumarase (FH).

To correlate the GABA accumulation after BTH treatments with the transcriptional activation, a qRT-PCR analysis of several genes involved in its biosynthesis (Figure 3) was carried out. Based on the described sequences in several species and using the Blast tool of Solgenomics database, we found the tomato orthologues of the citrate synthase (*mCS*), glutamate dehydrogenase (*GDH*), glutamate decarboxylase (*GAD*), glutamine synthetase (*GS*), aspartate aminotransferase (*AST*), and fumarase (*FH*) genes and designed specific oligonucleotide primers for qRT-PCR analysis (Supplementary Data Table S1).

**Figure 4:**
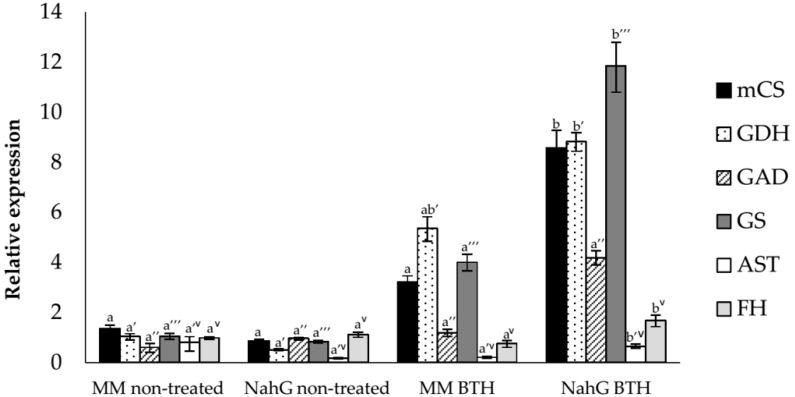
Expression levels of the tomato citrate synthase (mCS), glutamate dehydrogenase (GDH), glutamate decarboxylase (GAD), glutamine synthetase (GS), aspartate aminotransferase (AST), and fumarase (FH) genes in non-treated and BTH-treated (BTH) Money Maker and NahG mock tomato plants at 2.5 wpi, determined by real-time qRT-PCR analysis. Values were first normalized to the actine expression level. Expression levels are represented as mean ± standard error of three biological repetitions. An ANOVA analysis was performed for each gene, among the four samples (MM-non-treated, MM-BTH, NahG-non-treated and NahG-BTH). Different letters indicate the statistical significance differences among the four samples with *p*-value < 0.05.

The results of the induction of these genes in non-treated and BTH-treated Money Maker and NahG mock tomato plants are shown in Figure 4. All the transcripts were induced in Money Maker and NahG plants after the chemical treatments at 2.5 wpi, most of them statistically significant in the transgenic plants. These transcript inductions were well correlated with the relative GABA levels observed in NMR spectra in both genotypes after BTH treatments (Table 1).

### 3.3. Analysis of Primary Metabolites in Money Maker and NahG Tomato Plants after BTH Treatments

To identify other possible changes in primary metabolism underlying the BTH effect, an established gas cromatograpy-mass spectrometry (GC-MS) method was aplied to extracts from Money Maker and NahG tomato leaves after 48 h of 1 mM BTH treatments. A total of 20 amino acids, 22 organic acids, 19 sugars, 2 fatty acids, and 2 nucleotides were analyzed, and only GABA levels were statistically different after chemical treatments in both varieties. These results are in accordance with those obtained by the NMR platform (Table 1), confirming that GABA biosynthesis is induced by BTH in tomato plants.

**Figure 5:**
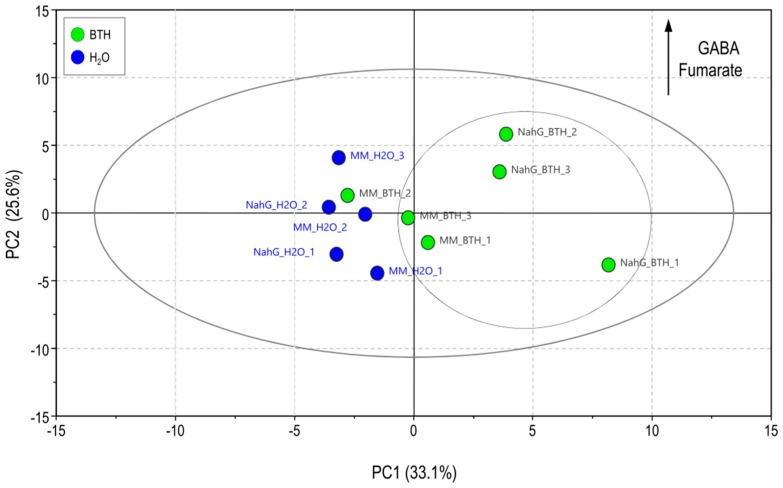
Score plot of principal component analysis (PCA) based on the characteristic ion of the mass spectra from the primary metabolites measured in the m/z range 35–900, of the Money Maker (MM) and NahG tomato plants after at 48 h after BTH treatments (green; leaves after BTH treatments; blue; leaves after H_2_O treatments).

Following the univariate statistical analysis of mass spectral data, a comparison of all the samples involved in the study was performed through a PCA analysis. An evident separation of BTH-treated NahG plants from all the others was observed by the score scatter plot of PCA, clearly showing that these transgenic plants undergo a greater metabolic change than its parental cultivar after chemical spray (Figure 5). In this case, the discriminant metabolites were GABA and fumarate which remained statistically over-accumulated in NahG tomato leaves after 48 h of BTH treatments. All of these results appear to indicate that GABA could play a role in the BTH-mediated recovery of NahG tomato plants infected with CEVd.

### 3.4. Effect of GABA Treatments in Money Maker and NahG Plants Infected by CEVd

**Figure 6:**
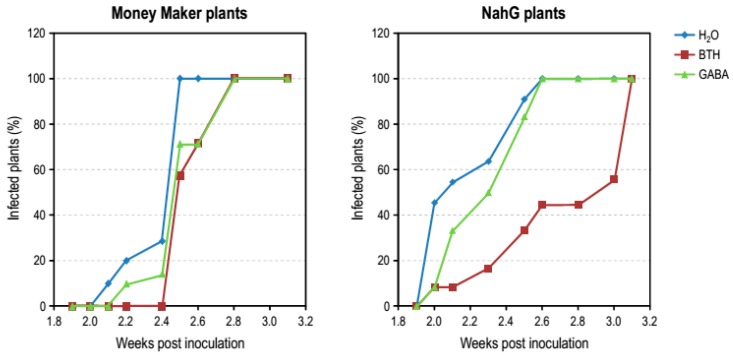
Disease development in Money Maker and NahG plants infected with CEVd and treated with BTH (red), GABA (green) or water (blue). Evolution of tomato plants showing symptoms at the indicated weeks post inoculation. Data correspond to one representative experiment.

To explore the implication of GABA in the BTH mechanism of action, Money Maker and NahG tomato plants infected by CEVd were pre-treated with 1 mM GABA, and a comparative study of the development of symptoms in CEVd-infected plants was carried out at the indicated time points. The number of tomato plants showing symptoms of the viroid disease was recorded during progression of infection. Applications of GABA in CEVd-inoculated Money Maker plants delayed the initial presence of symptoms as well as BTH (Figure 6, left panel). This effect was more evident in NahG transgenic plants (Figure 6, right panel). In fact, 2 weeks after viroid infection, the GABA protective effect was the same as that observed for BTH in NahG plants. However, a loss in the effectiveness of GABA treatments was observed over the time for both Money Maker and NahG tomato plants. Similar results were observed by analyzing symptom severity in NahG plants (Figure S1).

**Figure 7:**
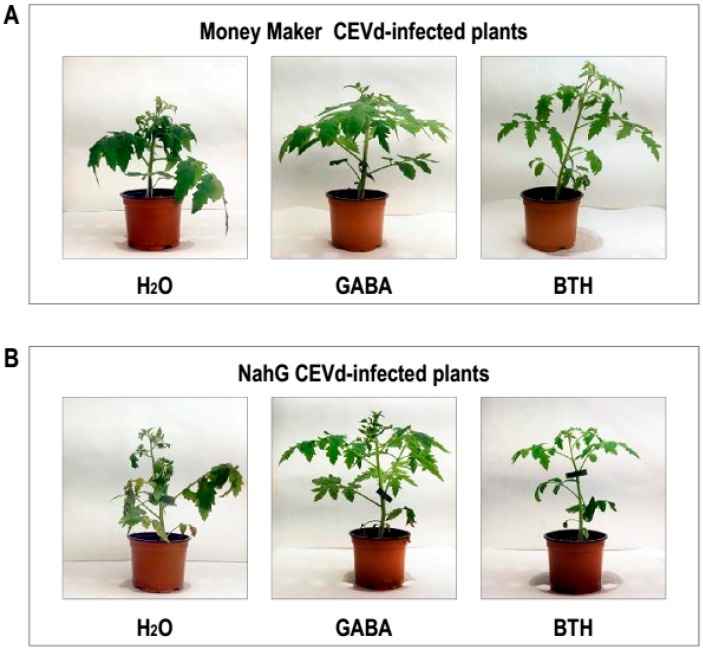
Growth of (**A**) Money Maker and (**B**) NahG plants following treatment with GABA or BTH and CEVd inoculation. Representative phenotype observed un infected plants 3 weeks after CEVd inoculation, and in equivalent plants pre-treated with 1 mM GABA or 1 mM BTH.

GABA treatment visibly reversed the enhanced susceptibility of NahG plants to CEVd during the early times of the infection, but its effectiveness along the entire study was lower as compared to BTH, thus indicating that other compounds added to GABA may be involved in the BTH action mechanism. Figure 7 depicts representative GABA and BTH restored phenotype of CEVd-infected MoneyMaker and NahG plants, respectively, at 3 weeks post inoculation.

### 3.5. Effect of GABA Treatments on the Expression Leves of PAL, PR1, and P23 Genes in Money Maker and NahG Plants

**Figure 8:**
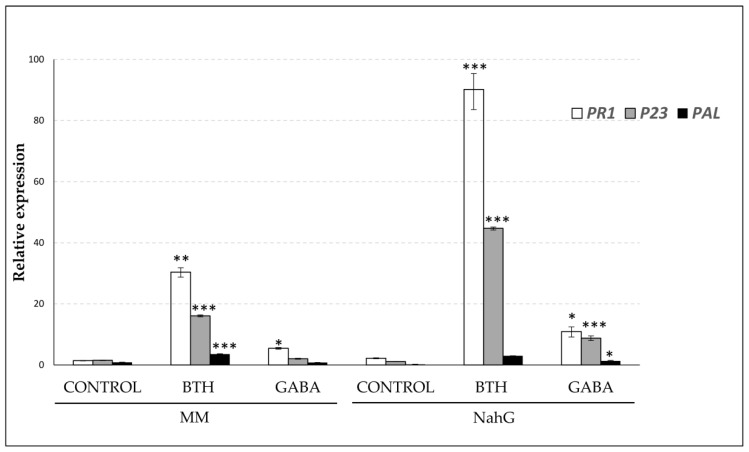
Expression levels of the tomato PAL, PR1 and P23 genes in Money Maker and NahG tomato plants, non-treated (CONTROL) and 48 h after BTH or GABA treatments. Values were first normalized to the actin expression level. Expression levels are represented as mean ± standard error of three biological repetitions. Asterisk (*), double asterisks (**) and triple asterisks (***) indicate significant differences between chemically-treated and non treated plants with *p* < 0.05, *p* < 0.01 and *p* < 0.001, respectively.

To better characterize the protective role of GABA observed in the first stages of the viroid infection, we explored the relative gene expression of PAL and the pathogenesis-related proteins PR1 and P23 [26] after 48 h of exogenous treatments with GABA. BTH treatments were used as a positive inducer of PR1 and P23 in tomato plants [5]. As Figure 8 shows, BTH clearly induced PR1, P23, and PAL genes in Money Maker plants, while only PR1 induction was statistically significant in the GABA-treated Money Maker plants. The pattern of induction of these genes was maintained in NahG plants, being the induction was more evident in the transgenic genotype. In fact, not only PR1, but also P23 and PAL were significantly induced in GABA-treated NahG plants, thus indicating that these transgenic plants could be more sensitive to BTH treatments.

## 4. Discussion

One of the main responses of plants to pathogen attack is the accumulation of defensive compounds, adapting the metabolome to the infection. Tomato plants effectively respond to different pathogens by synthesizing the appropriate metabolites to slow down the progress of the infection [17]. In this work, using the NMR metabolomics platform, we identified not only the compounds differentially accumulated in Money Maker and NahG tomato plants upon citrus exocortis viroid (CEVd) infection, but the metabolic adaptations induced by BTH treatments. The score plot of PLS based on the ^1^H-NMR data revealed evident metabolomic changes in these tomato plants after both viroid infection and BTH treatments (Figure 2A). While PC1 of PLS clearly explained the changes in the chemical composition following the viroid infection, PC2 described the metabolome after the BTH treatments. Moreover, this multivariate data analysis showed that the metabolome of NahG plants infected by CEVd was the most affected, which is in accordance to hyper susceptibility observed in these salicylic acid-deficient transgenic plants upon viroid infection [5]. Interestingly, when these plants were treated with BTH, both the phenotype and the metabolome were reestablished, placing them closer to their parental in the score plot. The score plots of both OPLS-DA performed upon the infection (Figure 2B) and the chemical treatment (Figure 2C) showed a clear separation indicative of the metabolic changes produced by CEVd and BTH, respectively.

In order to distinguish which metabolites were affected by each factor, an SUS correlation analysis was performed. The analysis of the positive size of the *y* axis of the SUS correlation showed the compounds accumulated in the viroid infection (Figure 2D). The defensive role of phenylpropanoids is well known, and the specific secondary response mediated by GA signaling is characteristic of the CEVd-tomato compatible interactions [17]. The induction of GA during systemic non-necrotizing infections, as those caused by CEVd, ToMV, and TSWV in tomato plants has been described in previous works [3,5]. In these compatible interactions GA acts as a pathogen-inducible signal of plant defenses and, in contrast with what occurs with SA, GA is predominantly conjugated to xylose [27]. Their precursor amino acids tyrosine and phenylalanine were also found to accumulate in the infected plants to supply the carbon skeleton needed for the biosynthesis of this phenolic compound. Furthermore, the increase in asparagine, aspartate, and glutamate levels have already been described in tomato plants upon ToMV inoculation [18].

To understand the mechanism of action of BTH, an analysis of the positive side of X axis of the SUS correlation was performed (Figure 2D). An increase in the levels of glutamine and BTH as well as its catabolyzed forms had already been found in BTH-treated Arabidopsis [12]. However the enhancement of GABA, adenosine, and trigonelline had not been described yet. Among them, GABA was the only compound that significantly accumulated after the chemical treatments in both Money Maker and NahG healthy tomato plants (Table 1), indicating that BTH could induce the biosynthesis of GABA.

On the other hand, in CEVd-infected tomato plants, BTH spray produced a statistically significant increase of several compounds, being not strictly correlated with the chemical treatment or with the infection process. Interestingly, in CEVd-infected BTH-treated NahG plants, phenylpropanoids accumulated as defence compounds despite the observed hyper susceptibility of these plants to this pathogen (Table 1). We postulate that this increase was not due to the chemical treatment, but to the viroid inoculation, since a reduction of the levels of these phenolic metabolites has been described in BTH-treated Arabidopsis plants [12]. The high levels of malic acid and glucose are needed to supply the substrates for the indispensable tricarboxylic acid cycle, while aspartate is required for the synthesis of alanine or threonine, and citric acid is the starting point for glutamine biosynthesis via α-ketoglutarate and glutamate (Figure 3).

Our results point to GABA as the lead compound highlighted by the NMR-metabolomics study in both genotypes of tomato plants after BTH-treatments. Since GABA levels were higher in the NahG plants than in their corresponding parental after both BTH spray (Table 1) or CEVd infection, SA could be impairing the GABA accumulation induced by this chemical. These results are in accordance with both the induction pattern of several genes involved in GABA biosynthesis, such as glutamate dehydrogenase (GDH), glutamate decarboxylase (GAD) or glutamine synthetase (GS) as analyzed by qRT-PCR (Figure 4), and the relative GABA levels measured by GC-MS 48 h post BTH treatments in Money Maker and NahG plants (See Section 3.3). The most effective BTH-treatment observed in NahG plants in terms of both GABA accumulation and transcriptional regulation of GABA biosynthetic genes could explain the remarkable reversion of the hyper susceptibility observed in these infected plants after BTH treatment. In this respect, we have observed that GABA treatments partially reverse the hyper susceptibility of NahG plants to CEVd (Figure 6, Figure 7) and trigger the induction of defense genes (Figure 8). Our results appear to unravel the GABA role in the BTH-mediated recovery of NahG tomato plants infected with CEVd.

GABA is a four-carbon non-protein amino acid, which acts in animals as an inhibitory neurotransmitter in the central nervous system. In plants, GABA concentrations increase rapidly in response to a number of stresses including heat, cold, salinity, drought, hypoxia, mechanical damage, and pathogens [28]. The role that GABA might play in plants ranges from an involvement in C and N metabolism, regulation of cytosolic pH, protection against oxidative stress, and mediation of interactions between plants and other organisms, including bacterial and fungal pathogens, nematodes and insects [29]. Both BTH and SA treatments result in increased production of reactive oxygen species (ROS) [30,31], and BTH inhibits catalase and ascorbate peroxidase, the two major H_2_O_2_-scavenging enzymes in plants [32]. Besides, overactivation of the GABA cycle has been described to result in resistance by both tightly controlling the defense-associated HR and slowing down the pathogen-induced senescence [22]. Therefore, the observed accumulation of GABA in BTH treated plants, and to a greater extent in NahG plants, could be related to a protection against ROS induced by BTH. Measures of ROS in BTH-treated NahG plants when compared to the corresponding BTH-treated parental Money Maker plants, could help to better correlate the GABA accumulation with the ROS production.

The protective role of GABA treatments has been described in several species subjected to different stresses. This metabolite has proven to alleviate oxidative damage in barley [33], to delay senescence in blueberry fruit by regulating the ROS response and phenylpropanoid pathway [34], and to induce resistance against *Penicillium expansum* by priming defense responses in pear fruit [20]. It had been demonstrated previously that exogenous treatments of GABA at similar concentrations used in this work, markedly promoted the induction of PAL in different tissue and plant organs [34,35,36]. This is in accordance with our results, since we have observed that GABA treatments induce PAL and other defensive genes such PR1 and P23 (Figure 8) mainly in NahG plants, producing a delay at the onset of symptom development in CEVd-infected tomato plants.

On the basis of our results, GABA can be considered as an additional signal to SA or GA involved in the induction of pathogenesis defence genes in tomato plants. In this context, it is worthy to state that genetic manipulation of GABA levels has demonstrated that GABA possesses a defensive role against drought and insect herbivory [37]. Additional studies are needed to further explore the effect of BTH on tomato plants related to GABA accumulation, in order to confirm the specific role of this metabolite in the defensive mechanisms triggered by BTH in plants.

## 5. Conclusions

BTH treatments produce quantitative and qualitative changes in the tomato metabolome, GABA being one of the most outstanding metabolites induced. This metabolic variation was clearly detectable using a NMR metabolomics platform. Interestingly, NahG tomato plants appear to be more sensitive to BTH or GABA treatments, in terms of both changes in metabolome and induction of defence genes. Our results suggest that the effects of GABA induction could be down-regulated by SA, since tomato plants impaired in the accumulation of this phenolic compound display a higher accumulation of GABA upon BTH treatment.

Taken together, our results indicate that GABA plays a role in tomato defense against CEVd, its protective action more effective in SA-defective NahG tomato plants. From the results presented here, it can also be hypothesized that the mechanism of action of BTH could be at least partially mediated by GABA.

## Figures and Tables

**Table 1:** Compounds significantly accumulated (*p*-value ≤ 0.05) after BTH treatments in mock or CEVd-inoculated Money Maker and NahG tomato plants with respect to non-treated corresponding plants.

	BTH Treated *vs* Non-Treated Plants	
	Money Maker	NahG
Mock-inoculated	GABA hydrolyzed-BTH	GABA ^1^ trigonelline
CEVd-inoculated	malic acid aspartic acid trigonelline	caffeic acid ^1^ ferulic acid ^1^ citric acid ^1^ malic acid ^1^ aspartic acid threonine alanine α-glucose trigonelline

^1^ Compounds differentially overaccumulated in BTH-treated NahG with respect to BTH-treated Money Maker tomato plants.

## Supplementary Materials

Supplementary materials can be found at https://www.mdpi.com/1999-4915/11/5/437/s1.

## References

[1] Mou Z., Fan W., Dong X. (2003). Inducers of Plant Systemic Acquired Resistance Regulate NPR1 Function through Redox Changes. Cell.

[2] Bellés J.M., Garro R., Pallás V., Fayos J., Rodrigo I., Conejero V. (2006). Accumulation of gentisic acid as associated with systemic infections but not with the hypersensitive response in plant-pathogen interactions. Planta.

[3] Bellés J.M., Garro R., Fayos J., Navarro P., Primo J., Conejero V. (1999). Gentisic acid as a pathogen-inducible signal, additional to salicylic acid for activation of plant defenses in tomato. Mol. Plant-Microbe Interact..

[4] Brading P.A., Hammond-Kosack K.E., Parr A., Jones J.D.G. (2000). Salicylic acid is not required for *Cf-2*- and *Cf-9*-dependent resistance of tomato to *Cladosporium fulvum*. Plant J..

[5] López-Gresa M.P., Lisón P., Yenush L., Conejero V., Rodrigo I., Bellés J.M. (2016). Salicylic Acid Is Involved in the Basal Resistance of Tomato Plants to Citrus Exocortis Viroid and Tomato Spotted Wilt Virus. PLoS ONE.

[6] Friedrich L., Lawton K., Ruess W., Masner P., Specker N., Rella M.G., Meier B., Dincher S., Staub T., Uknes S. (1996). A benzothiadiazole derivative induces systemic acquired resistance in tobacco. Plant J..

[7] Görlach J., Volrath S., Knauf-Beiter G., Hengy G., Beckhove U., Kogel K.H., Oostendorp M., Staub T., Ward E., Kessmann H. (1996). Benzothiadiazole, a novel class of inducers of systemic acquired resistance, activates gene expression and disease resistance in wheat. Plant Cell.

[8] Lawton K.A., Friedrich L., Hunt M., Weymann K., Delaney T., Kessmann H., Staub T., Ryals J. (1996). Benzothiadiazole induces disease resistance in Arabidopsis by activation of the systemic acquired resistance signal transduction pathway. Plant J..

[9] Benhamou N., Belanger R.R. (1998). Benzothiadiazole-mediated induced resistance to *Fusarium oxysporum* f. sp. *radicis-lycopersici* in tomato. Plant Physiol..

[10] Louws F.J., Wilson M., Campbell H.L., Cuppels D.A., Jones J.B., Shoemaker P.B., Sahin F., Miller S.A. (2001). Field Control of Bacterial Spot and Bacterial Speck of Tomato Using a Plant Activator. Plant Dis..

[11] Li X., Bi Y., Wang J., Dong B., Li H., Gong D., Zhao Y., Tang Y., Yu X., Shang Q. (2015). BTH treatment caused physiological, biochemical and proteomic changes of muskmelon (*Cucumis melo* L.) fruit during ripening. J. Proteom..

[12] Hien Dao T.T., Puig R.C., Kim H.K., Erkelens C., Lefeber A.W.M., Linthorst H.J.M., Choi Y.H., Verpoorte R. (2009). Effect of benzothiadiazole on the metabolome of Arabidopsis thaliana. Plant Physiol. Biochem..

[13] Vogt T. (2010). Phenylpropanoid biosynthesis. Mol. Plant.

[14] Katz V.A., Thulke O.U., Conrath U. (1998). A Benzothiadiazole Primes Parsley Cells for Augmented Elicitation of Defense Responses. Plant Physiol..

[15] Iriti M., Rossoni M., Borgo M., Faoro F. (2004). Benzothiadiazole Enhances Resveratrol and Anthocyanin Biosynthesis in Grapevine, Meanwhile Improving Resistance to Botrytis cinerea. J. Agric. Food Chem..

[16] Verpoorte R., Choi Y., Kim H. (2007). NMR-based metabolomics at work in phytochemistry. Phytochem. Rev..

[17] López-Gresa M.P., Maltese F., Bellés J.M., Conejero V., Kim H.K., Choi Y.H., Verpoorte R. (2010). Metabolic response of tomato leaves upon different plant-pathogen interactions. Phytochem. Anal..

[18] López-Gresa M.P., Lisón P., Kim H.K., Choi Y.H., Verpoorte R., Rodrigo I., Conejero V., Bellés J.M. (2012). Metabolic fingerprinting of Tomato Mosaic Virus infected Solanum lycopersicum. J. Plant Physiol..

[19] Shelp B.J., Bozzo G.G., Trobacher C.P., Zarei A., Deyman K.L., Brikis C.J. (2012). Hypothesis/review: Contribution of putrescine to 4-aminobutyrate (GABA) production in response to abiotic stress. Plant Sci..

[20] Yu C., Zeng L., Sheng K., Chen F., Zhou T., Zheng X., Yu T. (2014). γ-Aminobutyric acid induces resistance against Penicillium expansum by priming of defence responses in pear fruit. Food Chem..

[21] Bolton M.D. (2009). Primary metabolism and plant defense—Fuel for the fire. Mol. Plant Microbe Interact..

[22] Seifi H.S., Curvers K., de Vleesschauwer D., Delaere I., Aziz A., Hofte M. (2013). Concurrent overactivation of the cytosolic glutamine synthetase and the GABA shunt in the ABA-deficient sitiens mutant of tomato leads to resistance against Botrytis cinerea. New Phytol..

[23] Oldroyd G.E.D., Staskawicz B.J. (1998). Genetically engineered broad-spectrum disease resistance in tomato. Proc. Natl. Acad. Sci. USA.

[24] Roessner U., Wagner C., Kopka J., Trethewey R.N., Willmitzer L. (2000). Technical advance: Simultaneous analysis of metabolites in potato tuber by gas chromatography-mass spectrometry. Plant J..

[25] Bellés J.M., López-Gresa M.P., Fayos J., Pallás V., Rodrigo I., Conejero V. (2008). Induction of cinnamate 4-hydroxylase and phenylpropanoids in virus-infected cucumber and melon plants. Plant Sci..

[26] Conejero V., Bellés J.M., García-Breijo F., Garro R., Hernández-Yago J., Rodrigo I., Vera P., Fraser R.S. (1990). Signaling in Viroid Pathogenesis. Recognition and Response in Plant-Virus Interactions.

[27] Fayos J., Bellés J.M., López-Gresa M.P., Primo J., Conejero V. (2006). Induction of gentisic acid 5-*O*-β-D-xylopyranoside in tomato and cucumber plants infected by different pathogens. Phytochemistry.

[28] Kinnersley A.M., Turano F.J. (2000). Gamma Aminobutyric Acid (GABA) and Plant Responses to Stress. Crit. Rev. Plant Sci..

[29] Roberts M.R. (2007). Does GABA Act as a Signal in Plants?: Hints from Molecular Studies. Plant Signal. Behav..

[30] Kawano T., Sahashi N., Takahashi K., Uozumi N., Muto S. (1998). Salicylic Acid Induces Extracellular Superoxide Generation Followed by an Increase in Cytosolic Calcium Ion in Tobacco Suspension Culture: The Earliest Events in Salicylic Acid Signal Transduction. Plant Cell Physiol..

[31] Van der Merwe J.A., Dubery I.A. (2006). Benzothiadiazole inhibits mitochondrial NADH: Ubiquinone oxidoreductase in tobacco. J. Plant Physiol..

[32] Wendehenne D., Durner J., Chen Z., Klessig D.F. (1998). Benzothiadiazole, an inducer of plant defenses, inhibits catalase and ascorbate peroxidase. Phytochemistry.

[33] Song H., Xu X., Wang H., Wang H., Tao Y. (2010). Exogenous gamma-aminobutyric acid alleviates oxidative damage caused by aluminium and proton stresses on barley seedlings. J. Sci. Food Agric..

[34] Ge Y., Duan B., Li C., Tang Q., Li X., Wei M., Chen Y., Li J. (2018). γ-Aminobutyric acid delays senescence of blueberry fruit by regulation of reactive oxygen species metabolism and phenylpropanoid pathway. Sci. Hortic-Amsterdam.

[35] Ma Y., Wang P., Chen Z., Gu Z., Yang R. (2018). GABA enhances physio-biochemical metabolism and antioxidant capacity of germinated hulless barley under NaCl stress. J. Plant Physiol..

[36] Aghdam M.S., Kakavand F., Rabiei V., Zaare-Nahandi F., Razavi F. (2019). γ-Aminobutyric acid and nitric oxide treatments preserve sensory and nutritional quality of cornelian cherry fruits during postharvest cold storage by delaying softening and enhancing phenols accumulation. Sci. Hortic-Amsterdam.

[37] Bown A.W., Shelp B.J. (2016). Plant GABA: Not Just a Metabolite. Trends Plant Sci..

