# Supplementary material for: Effect of Benzothiadiazole on the Metabolome of Tomato Plants Infected by Citrus Exocortis Viroid
Source: Viruses. 2019 May 14;11(5):437. doi: 10.3390/v11050437 (PMC6563216; doi:10.3390/v11050437)
Supplement: Supplementary file 1 [file viruses-11-00437-s001.pdf]

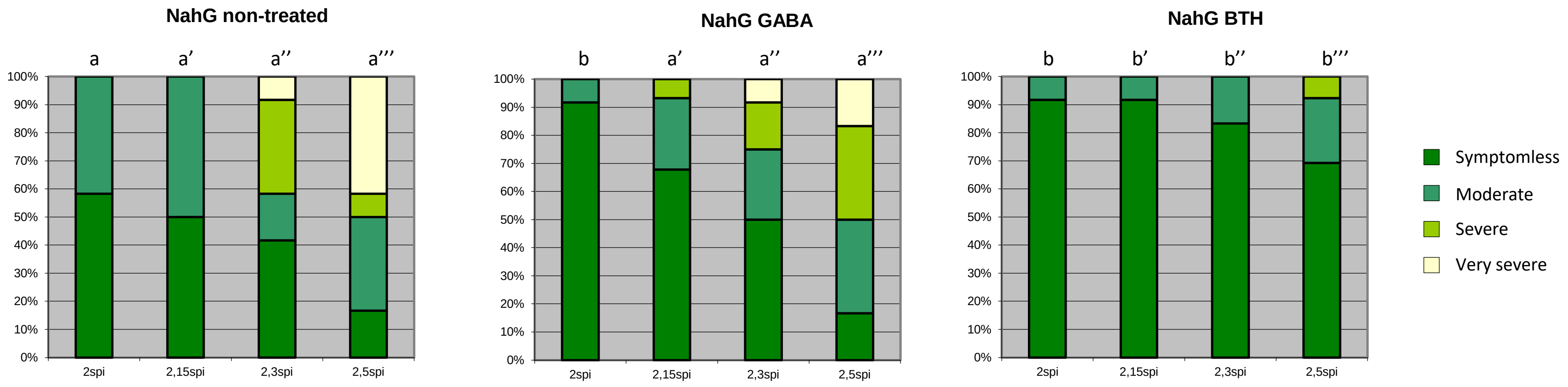

Figure S1. Disease severity of CEVd-infected NahG tomato plants treated with BTH or GABA with respect to the corresponding non-treated plants. Symptomatology was scored at 2, 2.15, 2.3 and 2.5 weeks post-inoculation (wpi) using the following scale: symptomless, moderate, severe, and very severe. Data of a representative experiment are shown. A Kruskal-Wallis analysis was performed and different letters indicate significant differences ($p<0.05$) between the non-treated and chemical treated NahG tomato plants.
